# Pentathiepins are an understudied molecular prism of biological activities

**DOI:** 10.1002/ardp.202400646

**Published:** 2024-10-09

**Authors:** Luca Pozzetti, Christopher R. M. Asquith

**Affiliations:** ^1^ School of Pharmacy, Faculty of Health Sciences University of Eastern Finland Kuopio Finland

**Keywords:** pentathiepin, polysulfide, sulfur, TC‐2153, varacin

## Abstract

The pentathiepin core was first synthesized in 1971, and while synthetic techniques have progressed over subsequent decades, the biological applications of this heterocycle have received less attention and are only now becoming more apparent. The first natural product, varacin, was identified in 1991, showing cytotoxicity toward a human colon cancer cell line. More recently, the pentathiepin has acted as a surrogate to replace elemental sulfur, that was discovered as a hit in neurodegenerative animal models. A variety of other medicinal chemistry applications have recently been disclosed. Here, we summarize these indications and highlight the main synthetic pathways to access the pentathiepin core. We offer a concise summary and future perspective of this unique sulfur isosteric replacement.

## Graphical abstract

Pentathiepins are cyclic sulfur‐rich compounds that possess remarkable chemical stability, unique structural features, and an extraordinary variety of biological activities. Nonetheless, they have only drawn the attention of the scientific community on a few occasions, generally after the serendipitous discovery of some of their properties. This review aims to highlight the wealth opportunities this understudied heterocycle has to offer.

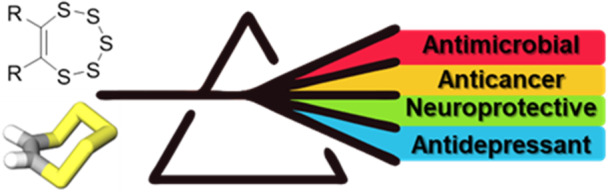

## 1 INTRODUCTION

**Figure 1:**
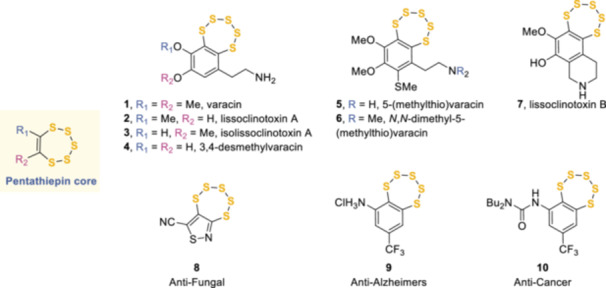
Natural and several key synthetic biologically active pentathiepins.

Pentathiepins (in the literature both spellings, “pentathiepine” and “pentathiepin”, are present, whereas the IUPAC nomenclature recommends “pentathiepine”; in this review, we will use “pentathiepin” to be consistent with the terminology used in previous landmark publications^[^ ^1^, ^2^, ^3^ ^]^) are a class of seven‐membered ring organic compounds containing a carbon–carbon double bond and five sulfur atoms (Figure 1). Even though the first reported synthesis of a similar, fully saturated, ring dates back to 1967,^[^ ^4^ ^]^ the interest in this unusual core peaked with the discovery of the cytotoxic natural product varacin (**1**) in 1991 by Ireland et al.^[^ ^3^ ^]^ from *Lissoclinum vareau*, a marine organism found in the Fiji Islands. The subsequent discovery of further varacin analogs, namely lissoclinotoxin A (**2**), isolissoclinotoxin A (**3**), 3,4‐desmethylvaracin (**4**), 5‐(methylthio)varacin (**5**), *N*,*N*‐dimentyl‐5‐(methylthio)varacin (**6**), and lissoclinotoxin B (**7**), in 1994^[^ ^5^, ^6^ ^]^ created a distinct group of organic natural products with an extremely high percentage of sulfur. These compounds all showed interesting biological activities, ranging from cytotoxicity against cancer cell lines to protein kinase C (PKC) inhibition and bactericidal properties. Previous to these disclosures, researchers at E. I. Du Pont de Nemours developed the first synthetic pentathiepin (**8**), which displayed potent antifungal activity.^[^ ^7^, ^8^, ^9^, ^10^ ^]^ These promising biological activities prompted chemists to devise several synthetic routes to pentathiepins involving different sulfur introduction mechanisms, as extensively reviewed by Konstantinova et al. in 2004.^[^ ^2^ ^]^ A period of relative dormancy followed until pentathiepin **9**, also known as TC‐2153, was discovered as a potent inhibitor of striatal‐enriched protein tyrosine phosphatase (STEP), which has an indication in neurodegenerative diseases.^[^ ^11^ ^]^ Another compound, **10**, a structural analog of **9**, also showed promising anticancer activity targeting tyrosyl‐DNA phosphodiesterase 1 (TDP1).^[^ ^12^ ^]^ To the best of our knowledge, the only reviews of the pentathiepins class were published in 2004^[^ ^9^ ^]^ and an update in 2007,^[^ ^12^ ^]^ despite several significant developments in both the synthesis and the applications of these heterocycles. We have collected comprehensive information regarding synthetic routes, physical properties, and biological applications of pentathiepins, with particular emphasis on the most recent advances.

## 2 SYNTHESES OF PENTATHIEPINS

### 2.1 Initial syntheses

**Scheme 1:**
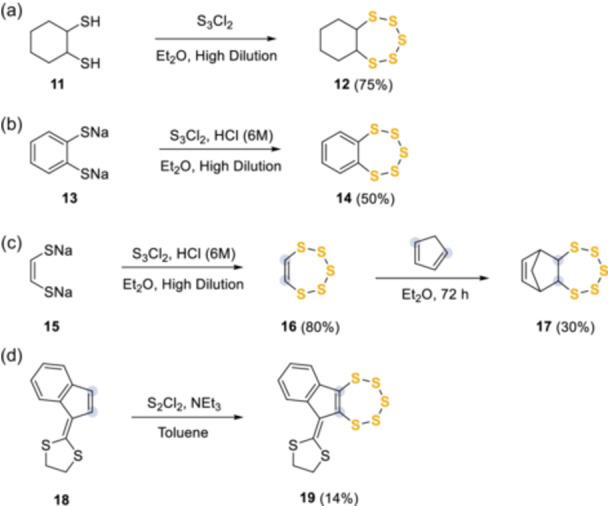
First synthetic approaches using S_3_Cl_2_ and S_2_Cl_2_.

The impressive array of biological activities has encouraged the development of further synthetic routes to find quick and effective routes to access the chemical space of the pentathiepin scaffold. Several general methods have been developed, some of which are both robust and high‐yielding; however, functional group tolerance is often a limitation. To combat this most methods are based on the introduction of sulfur at an early stage in the process to minimize complications of a late‐stage introduction. The first reported synthesis of a seven‐membered ring containing five connected sulfur atoms and two connected carbons^[^ ^4^ ^]^ involved the conversion of a simple *trans*−1,2‐cyclohexanedithiol **11** into hexahydrobenzo[*f*][1,2,3,4,5]pentathiepin **12**, by exploiting dichlorotrisulfane (S_3_Cl_2_), as sulfur source (Scheme 1a). Over the proceeding decades, this fully saturated pentathiepin class received much less attention compared with their unsaturated counterparts, partially due to synthetic challenges and a lack of natural products and known biological activities.^[^ ^13^ ^]^ Indeed, following the early success of this work, Fehér et al.,^[^ ^1^ ^]^ starting from the sodium salts **13** to stabilize the thiol groups, reported the first example of a benzo[*f*][1,2,3,4,5]pentathiepin core (**14**) (Scheme 1b). The authors were also able to convert the dithiol **15** into 1,2,3,4,5‐pentathiepin **16** and to exploit its high sulfur content (86%) as dienophile in a Diels–Alder reaction to give **17** in moderate yield (Scheme 1c). A few years later, Ariyan et al.^[^ ^14^ ^]^ were the first to employ the more readily available and stable disulfur dichloride (S_2_Cl_2_), by reaction with 2‐(1*H*‐inden‐1‐ylidene)−1,3‐dithiolane **18** to obtain **19** (Scheme 1d). The authors proposed an initial elimination of two moles of HCl, followed by a ring‐closing elimination of sulfur dichloride and the formation of the 7‐membered ring, but the full reaction mechanism has not been defined.

**Scheme 2:**
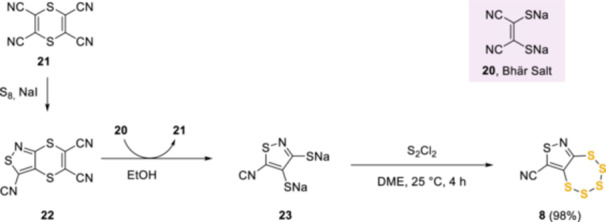
Synthesis of the antifungal pentathiepin **8.**

This was followed by researchers at E. I. Du Pont de Nemours who discovered [1,2,3,4,5]pentathiepino[6,7‐*c*]*iso*thiazole‐8‐carbonitrile **8** as an antifungal agent in 1977.^[^ ^7^ ^]^ To access this pentathiepin **8**, an innovative synthetic methodology utilizing Bhär's salt (**20**)^[^ ^15^ ^]^ was used to mask intermediate **22**, obtained from 1,4‐dithiine‐2,3,5,6‐tetracarbonitrile **21** (Scheme 2). The idea was based on earlier work^[^ ^16^ ^]^ that showed **22** was thermodynamically stable and its formation would act as a thermodynamic sink. The use of Bhär's salt to mask this intermediate enabled ready access to the pentathiepin precursor **23**, which was in turn converted into pentathiepin **8** by treatment with S_2_Cl_2_ in 1,2‐dimethoxyethane (DME) in almost quantitative yield.^[^ ^17^ ^]^

**Scheme 3:**
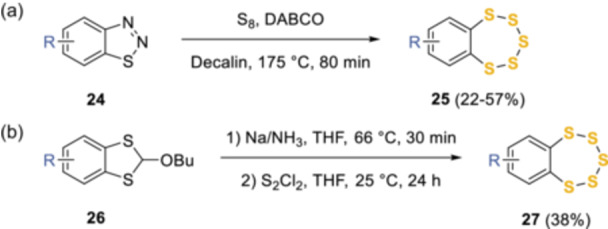
Mid‐1980s syntheses by Chenard and colleagues.

Subsequently, in the mid‐1980s Chenard et al. adopted a general method to prepare benzopentathiepins **25** involving the substitution of the 1,2,3‐thiadiazole functionality (**24**) using relatively harsh conditions, boiling with elemental sulfur in decalin, in the presence of one equivalent of 1,4‐diazabicyclo[2.2.2]octane (DABCO) (Scheme 3a).^[^ ^18^ ^]^ This route was also extended with moderate yields to other heterocyclic fused rings including 1,2,3‐thiadiazoles or 1,2,3‐selenodiazoles.^[^ ^19^, ^20^ ^]^ A second method involved the construction of an alkoxybenzodithiole (**26**, Scheme 3b) by exploiting the benzyne reaction with CS_2_ and butanol; the subsequent reduction to vicinal dithiol with sodium in ammonia, followed by the addition of S_2_Cl_2_ resulted in the final construction of the pentathiepin ring **27**.^[^ ^21^ ^]^

**Scheme 4:**
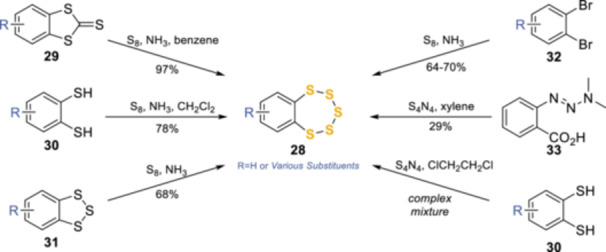
Synthesis evolution toward a common approach to the benzopentathiepin core.

Subsequently, several different methods were developed to the general structure exemplified by **28** (Scheme 4) building on these early approaches, enhancing both the reliability and yield. Sato et al. have made a significant contributions to the pentathiepin functional group synthesis routes and biological evaluation. The first route developed was the utilization of a bridging thiocarbonyl, forming a 1,3‐dithiole‐2‐thione (**29**) and corresponding treatment with sulfur and ammonia in benzene. Interestingly, the same conditions could be applied to the dithiol **30**, which is considered to be an intermediate of this reaction, but this led to a ~20% reduction in yield.^[^ ^22^, ^23^ ^]^ The second and third routes used the same conditions with trithiole **31**, or dibromo **32** ^[^ ^24^, ^25^, ^26^ ^]^ starting materials. Morris and Rees^[^ ^27^ ^]^ also focused extensively on high percentage sulfur and nitrogen heterocyclic systems. Initial work focused on the (*E*)‐2‐(3,3‐dimethyltriaz‐1‐en‐1‐yl)benzoic acid **33** and tetrasulfide tetranitride, a binary sulfide containing only sulfur and nitrogen in an extreme cradle conformation.^[^ ^28^ ^]^ The use of tetrasulfide tetranitride resulted in the production of pentathiepins in modest yield; however, attempts to apply the same conditions to **30** yielded an inseparable complex mixture including a tetrathiazepine derivative.^[^ ^27^ ^]^

### 2.2 Route to the total synthesis of varacin

**Scheme 5:**
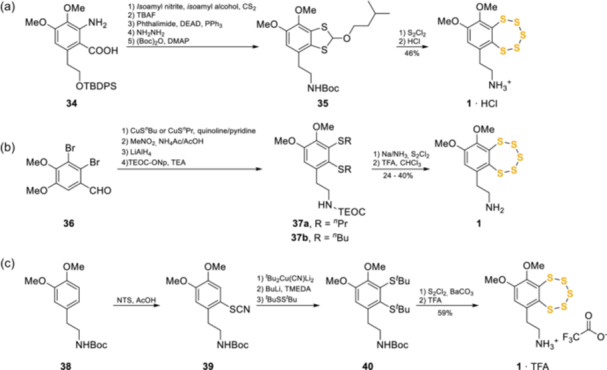
Syntheses of varacin **1**.

The discovery of varacin and its analogs prompted the development of several different routes to achieve the total synthesis (Scheme 5a–d). One of the first methods, reported by Behar et al. in 1993,^[^ ^29^ ^]^ started from the properly substituted 2‐*iso*amyloxybenzo[*d*][1,3]dithiole **35**, which was in turn treated by following the previous procedure developed by Chenard et al.,^[^ ^21^ ^]^ with minor alterations. This was followed by Ford et al. who relied on the insertion of vicinal alkylated dithiols onto the aromatic ring as sulfides by nucleophilic substitution of an aromatic dibromo precursor (**36**) with cuprous alkylmercaptide in quinoline/pyridine at 160°C.^[^ ^30^, ^31^ ^]^ A different approach was developed by Toste et al. in 1995, which relied upon the introduction of only a single sulfur substituent, by a relatively mild electrophilic reaction. In particular, Boc‐protected commercially available **38** underwent a regioselective electrophilic thiocyanation procedure followed by conversion to *tert*‐butyl sulfide **39**. Lithiation with butyl lithium and reaction with di‐*tert*‐butyl disulfide afforded the key intermediate **40**, which was then subjected to the construction of the pentathiepin ring by using S_2_Cl_2_ with the addition of barium carbonate (BaCO_3_) to increase the yields and reduce the formation of other polysulphide contaminants.^[^ ^32^ ^]^

### 2.3 Progression in routes to access the benzopentathiepin core

**Scheme 6:**
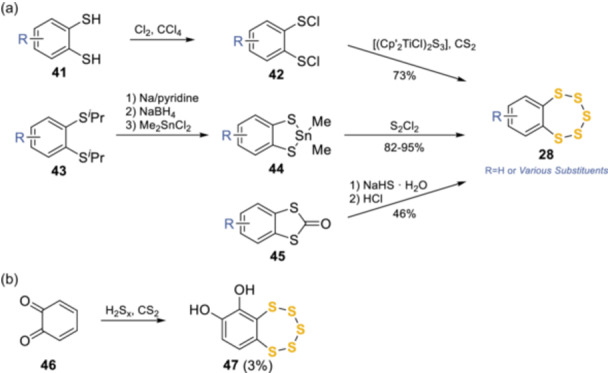
Recent syntheses of the pentathiepin core.

As the field progressed into the 2000s, several other synthetic routes were developed to access the benzopentathiepin core **28** (Scheme 6a). These included the functionalizing of vicinal dithiol **41** to sulfenyl chloride **42** and treating this using the titanocene complex [(Cp’_2_TiCl)_2_S_3_] to form **28** in 73% yield by Steudel et al.^[^ ^33^ ^]^ Another developed by Sato et al. involved precoordinating dichlorodimethylstannane to vicinal alkylated dithiols **43** to produce **44**, which can then be treated with disulfur dichloride to furnish **28** in high yield.^[^ ^34^, ^35^ ^]^ To remove the need for aggressive conditions, Khomenko et al.^[^ ^36^, ^37^ ^]^ treated dithiolone **45** with sodium hydrosulphide hydrate in DMSO followed by hydrochloric acid. This procedure afforded the desired pentathiepin in a moderate 46% yield but avoided the use of a metal solution. Aebisher et al. investigated the reaction between *o*‐benzoquinone **46** and polysulphides H_2_S_*x*_,^[^ ^38^ ^]^ as models for the possible 2‐electron transfer reaction between dopamine‐*o*‐quinone and these reactive sulfur species (Scheme 6b) with the aim of understanding the possible biosynthetic origin of natural benzopentathiepins. Despite this protocol yielding pentathiepin **47** only in low yield together with several other byproducts, it was the first reported attempt to study the mechanisms underlying the biosynthesis of the pentathiepin sulfur‐rich natural products.^[^ ^38^ ^]^

### 2.4 Access to other pentathiepins

**Scheme 7:**
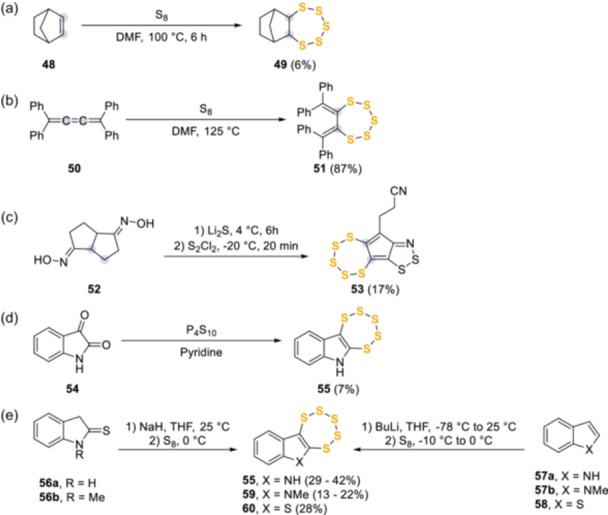
C–H activated synthetic routes to access novel pentathiepin scaffolds.

Several other synthetic routes have been developed which involved the construction of the pentathiepin ring across carbon–carbon bonds (Scheme 7a‐e). The genesis of this approach was initially reported by Bartlett et al.^[^ ^39^ ^]^ showedg the first more generalized C–H activated approach by treatment of **48** with refluxing elemental sulfur in DMF to form hexahydro‐6,9‐methanobenzo[f][1,2,3,4,5]pentathiepin **49** (Scheme 7a). This idea was followed by investigations into the reactivity of 1,1,4,4‐*tetra*phenylbuta‐1,2,3‐triene **50** with elemental sulfur and selenium by Ando et al.^[^ ^20^, ^40^ ^]^ (Scheme 7b). This resulted in two different outcomes where both chalcogens were not analogous, sulfur resulted in **51**, but selenium preferentially forms an unsymmetrical dimer. This approach also demonstrated that a C–H activated mechanism can deliver **51** and related analogs in a high yield, albeit from a relatively electron‐rich starting material. The formation of 3‐([1,2,3,4,5]pentathiepino[6’,7’:3,4]cyclopenta[1,2‐*d*][1,2,3]dithiazol‐9‐yl)propanenitrile **53** from a domino cascade reaction was another key paper by Rees et al.^[^ ^41^ ^]^ as this work demonstrated potential for a dual domino sulfur functionality creation. This innovative cascade reaction from the di‐oxime **52** to form **53** while relatively poor yielding, allowed access to a highly functionalized product from a relatively simple starting material (Scheme 7c). Then Bergman et al.^[^ ^42^ ^]^ utilized diphosphorous pentasulphide dissolved in pyridine as a sulfur source to form 6*H*‐[1,2,3,4,5]pentathiepino[6,7‐*b*]indole **55** from isatin **54** (Scheme 7d). This method consumed a double functionality and was low yielding even after optimization, but it catalyzed further work to develop a higher yield approach from less functionalized starting material. This was achieved by treating indoline‐2‐thiones **56a**,**b** with sodium hydride followed by elemental sulfur to produce the corresponding pentathiepins **55** and **59** (13%–42%)^[^ ^43^ ^]^ and by treating indoles **57a**,**b** with butyl lithium and elemental sulfur (22%–29%). Interestingly, the same conditions applied to benzo[*b*]thiophene **58** afforded the analogous **60** in 28% yield (Scheme 7e).^[^ ^44^ ^]^

### 2.5 S_2_Cl_2_/DABCO methodology development

**Scheme 8:**
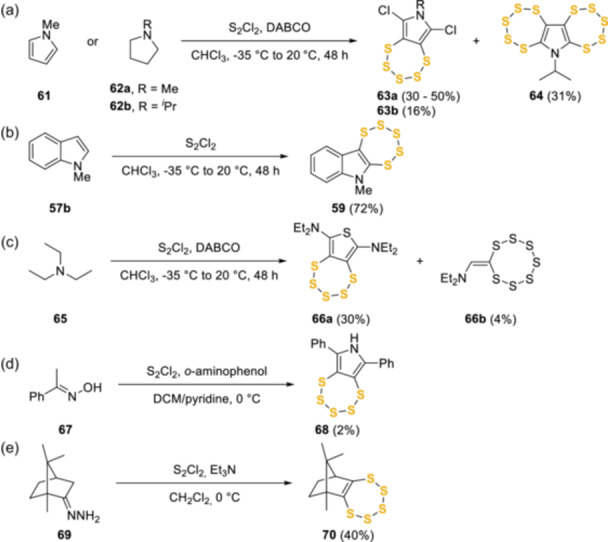
Application of S_2_Cl_2_ to the synthesis of pentathiepin core.

In the early 2000s, Rakitin and Rees et al. demonstrated a one‐step protocol to form fused pentathiepins with direct addition of sulfur *via* S_2_Cl_2_ in some cases combined with a variety of pyrroles (**61**), pyrrolidines (**62**), and indole derivatives (**57b**) (Scheme 8a,b). S_2_Cl_2_ formed an integral part of the reaction acting as both a sulfur and, in some cases, an oxidation and chlorination source to produce examples such as 6,8‐dichloro‐7‐methyl‐7*H*‐[1,2,3,4,5]pentathiepino[6,7‐*c*]pyrrole **63a**, in which the pentathiepin moiety was added to the pyrrole ring at the γ‐position, and both α‐positions were chlorinated. Interestingly, compound **64**, obtained as an unexpected major product from *N*‐isopropylpyrrolidine **62b**, was the first *bis*pentathiepin example reported.^[^ ^45^, ^46^ ^]^

Subsequent to the discovery of the DABCO‐S_2_Cl_2_ mediated approach to activate the otherwise inert Hünig's base,^[^ ^47^ ^]^ this complex was utilized in the creation of other remarkable compounds in one‐pot multiple sequence reactions including *bis*[1,2]dithiolo[1,4]thiazines,^[^ ^48^ ^]^ and *bis*[1,2]dithiolopyrroles.^[^ ^49^ ^]^ Rakitin and Rees et al. also reported the synthesis of other diverse compounds including the unprecedented conversion of triethylamine **65** and S_2_Cl_2_ into a thienopentathiepin **66a** and a heptathiocane **66b** (Scheme 8c).^[^ ^50^ ^]^ The versatility of disulfur dichloride in the synthesis of pentathiepins from a wide variety of starting materials has also been demonstrated by reports of acetophenone oxime (**67**) conversion to pyrrolepentathiepin **68** in the presence of pyridine and *o*‐aminophenol, albeit in very low yield (2%) as a side‐product.^[^ ^51^ ^]^ The related synthesis of camphor‐derived pentathiepin **70** from the corresponding hydrazone **69** with S_2_Cl_2_ in the presence of triethylamine was achieved in a 40% yield.^[^ ^52^ ^]^

### 2.6 Recent synthetic developments

**Scheme 9:**
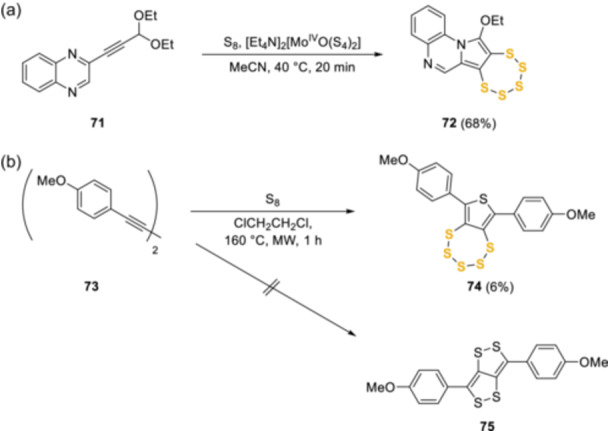
More recent synthetic approaches to the pentathiepin core**.**

More recently, the chemical space and routes to the pentathiepin has significantly expanded. These include the use of a dithiolene‐derived molybdenum complex approach with elemental sulfur developed by Schulzke et al. for the conversion of activated alkynes, such as **71**, to unsymmetrical pentathiepin, such as **72**, in a cascade reaction (Scheme 9a).^[^ ^53^, ^54^ ^]^ The approach appears to be quite substrate‐specific, but with short reaction times, mild conditions, and a high yield, it marks a significant milestone in the synthetic progression. It also demonstrates that the pentathiepin ring is both stable and can be isolated without major impurities. Another example of the pentathiepin stability is in the undesired construction of **74** from the mildly activated alkyne **73**, during the investigation into dithiolodithiole compounds. Schipper et al.^[^ ^55^ ^]^ developed a robust synthetic route (Scheme 9b), but were unable to isolate the corresponding 3,6‐*bis*(4‐methoxyphenyl)‐[1,2]dithiolo[4,3‐*c*][1,2]dithiole **75**, obtained in a complex mixture together with 6,8‐*bis*(4‐methoxyphenyl)thieno[3,4‐*f*][1,2,3,4,5]pentathiepin **74**, which was purified (6% yield) and characterized.

## 3 REACTIVITY OF PENTATHIEPINS

**Scheme 10:**
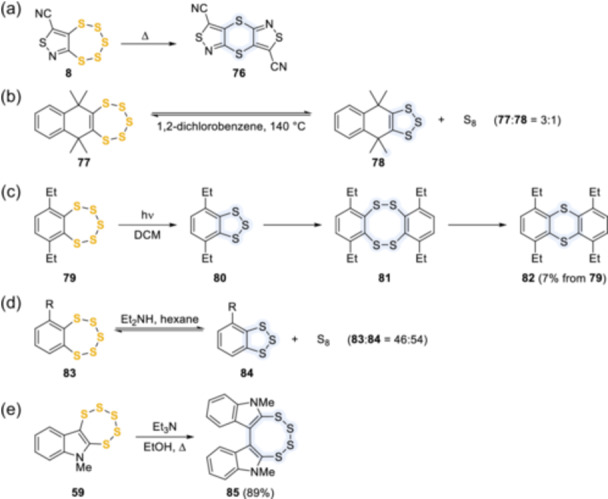
Desulfurization of pentathiepin rings due to thermolysis, photolysis, and treatment with various amine bases.

Bicyclic and tricyclic pentathiepins have both been shown to possess varied chemical properties, involving not only reactivity at the S_5_ ring, which most commonly leads to partial or total desulfurization but also reactions at the attached ring or its substituents, thus demonstrating the stability of the pentathiepin ring to a variety of conditions. Many of these reactions have been discovered or developed during the various attempts made by chemists to produce the pentathiepin ring itself. As shown in Scheme 10, heating pentathiepin **8** afforded 1,4‐dithiin **76**,^[^ ^17^ ^]^ while heating **77** gave a mixture of the starting material, 1,2,3‐trithiole **78**, and sulfur.^[^ ^56^ ^]^ Similarly, the irradiation of diethylbenzopentathiepin **79** in dichloromethane with medium‐ or high‐pressure mercury lamp resulted in subsequent desulfurization to trithiole **80**, dimerization to tetrathiocin **81**, and final ring contraction to 1,4‐dithiin **82**.^[^ ^57^ ^]^ Desulfurization of pentathiepins has also been reported in the presence of secondary or tertiary amines. *o*‐Substituted benzopentathiepin **83** lost two sulfur atoms in the presence of diethylamine in hexane, affording trithiole **84** ^[^ ^21^ ^]^; whereas pentathiepinoindole **59** gave tetrathiocin **85** in 89% yield.^[^ ^43^ ^]^

**Scheme 11:**
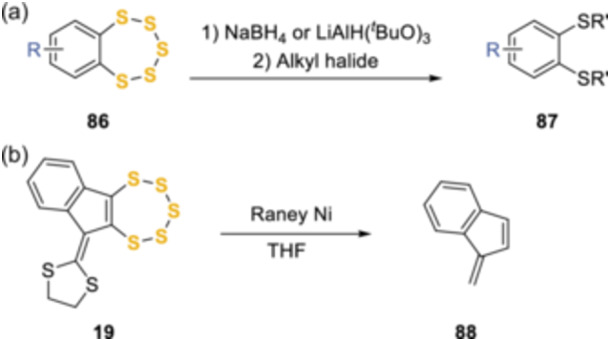
Instability of the pentathiepin ring to reducing agents.

**Scheme 12:**
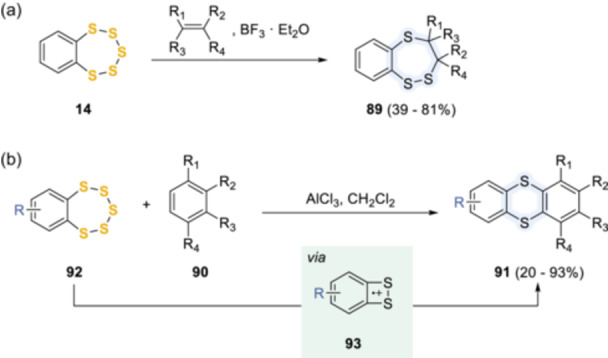
Reactivity of pentathiepins with Lewis acids.

**Scheme 13:**
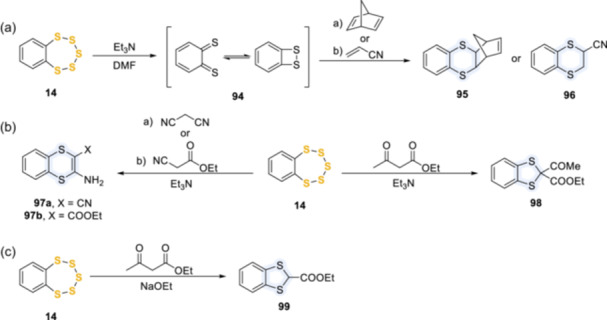
Reaction of pentathiepins in the presence of triethylamine and other bases.

**Scheme 14:**
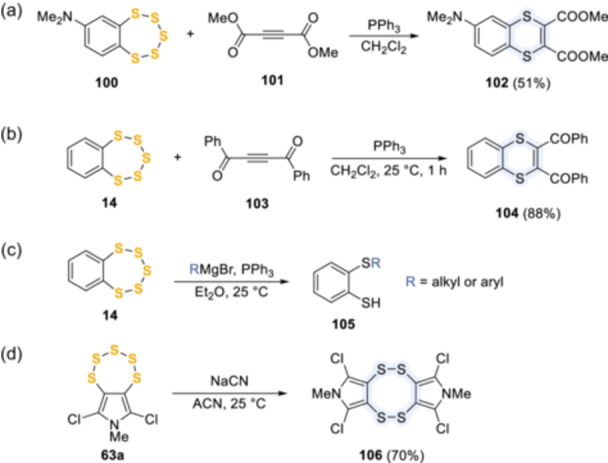
Reaction of pentathiepins in the presence of triphenylphosphine.

The pentathiepin ring is sensitive to reducing agents, including sodium borohydride and lithium tri(*tert*‐butoxy)aluminum hydride, leading to the *bis*dithiol formation, which can then be alkylated in situ (Scheme 11). A complete desulfurization can also take place by treatment of pentathiepins with Raney® nickel.^[^ ^3^, ^6^, ^14^, ^24^, ^58^ ^]^ In the presence of Lewis acids (Scheme 12), triethylamine (Scheme 13), or triphenylphosphine (Scheme 14), the pentathiepin ring underwent various desulfurization pathways that could be trapped by the addition of alkenes, alkynes, aromatic compounds, Grignard reagents, or active methylene compounds such as malononitrile, ethyl cyanoacetate, ethyl acetoacetate, acetylacetone, and ethyl 2‐chloropropionate. As shown in Scheme 12, when catalyzed by boron trifluoride etherate, the unsymmetrical 1,2,5‐benzotrithiepins **89** were formed where the pentathiepin reacted as a 1,5‐dipole equivalent.^[^ ^59^ ^]^ While benzopentathiepins **92** afforded the corresponding thianthrenes **91** when treated with different aromatic rings in the presence of AlCl_3_.^[^ ^60^ ^]^ These Friedel–Crafts‐type reactions could proceed through a 1,2‐benzodithiete radical cation (**93**), as observed by Bock et al.^[^ ^61^ ^]^

In the presence of triethylamine, benzopentathiepin **14** opened and lost three sulfur atoms, generating intermediate **94**, which in turn afforded 1,4‐dithianes **95** and **96** in the presence of the respective alkenes (Scheme 13a).^[^ ^62^ ^]^ Similarly, triethylamine determined the transformation of **14** into 1,4‐dithiins **97a** and **97b** in the presence of malononitrile and ethyl cyanoacetate, respectively; while the treatment with ethyl acetoacetate led to benzodithiole **98** (Scheme 13b). Interestingly, the use of sodium ethoxide instead of triethylamine afforded benzodithiole **99** (Scheme 13c).^[^ ^63^ ^]^

Similarly, the nucleophilic attack by triphenylphosphine or sodium cyanide led to the opening of the pentathiepin ring and to the formation of intermediate **94** (or a derivative thereof), which could be intercepted by dimethyl acetylenedicarboxylate (DMAD) **101** to yield 1,4‐dithiin **102**,^[^ ^21^, ^50^ ^]^ dibenzoylacetylene **103** to afford **104**,^[^ ^64^ ^]^ or Grignard reagents, to give 2‐alkylthio‐ and 2‐arylthiobenzenethiols **105** ^[^ ^65^ ^]^ (Scheme 14a‐c). Interestingly, the reaction of **63a** with three equivalents of NaCN in acetonitrile at ambient temperature also gave the stable byproduct **106**, whose yield could be improved up to 70% by high dilution (Scheme 14d).^[^ ^66^, ^67^ ^]^

**Scheme 15:**
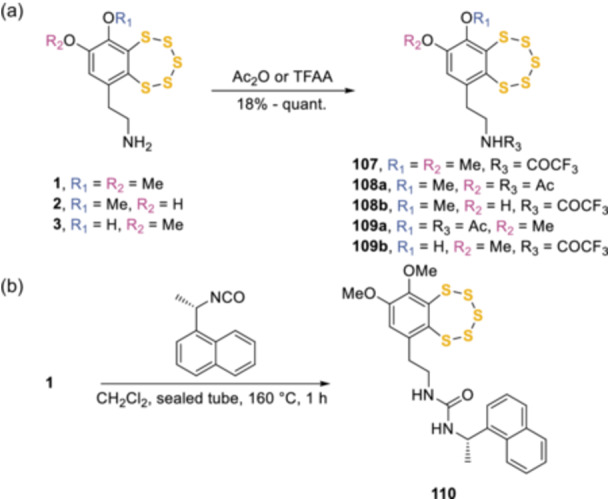
Protection of varacin and its natural analogs.

The susceptibility of pentathiepin rings to nucleophiles is widely recognized and has been demonstrated in a context related to the biological mechanism of action of varacin and related analogs.^[^ ^68^ ^]^ The protection of the lateral‐chain amino group of this class of natural compounds has been of particular interest and has been addressed by several different functional groups (Scheme 15). The *N*‐acetylation and trifluoroacetylation have been reported with acetic^[^ ^5^, ^69^ ^]^ and trifluoroacetic anhydrides^[^ ^31^, ^70^ ^]^ (Scheme 15a) on varacin (**1**), lissoclinotoxin A (**2**), and isolissoclinotoxin A (**3**), together with the conversion of **1** into a urea moiety (**110**) with (*S*)‐(+)‐1‐(1‐naphthylethyl) isocyanate (Scheme 15b).^[^ ^71^ ^]^ Pentathiepins proved to be stable also in various deprotecting steps, in the presence of hydrochloric or trifluoroacetic acids.^[^ ^29^, ^31^ ^]^

**Scheme 16:**
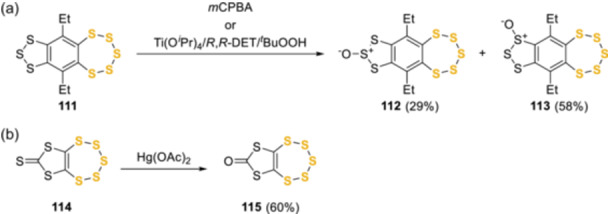
Stability of the pentathiepin ring toward oxidations.

The stability of pentathiepin rings toward oxidative conditions was demonstrated by the selective oxidation of the trithiole ring of **111** in the presence of *m*‐chloroperbenzoic acid (*m*CPBA)^[^ ^72^ ^]^ or Sharpless reagent,^[^ ^73^ ^]^ and by the conversion of thioketone **114** into ketone **115** with mercuric acetate^[^ ^74^ ^]^ (Scheme 16a,b).

## 4 STRUCTURAL PROPERTIES OF PENTATHIEPINS

**Scheme 17:**
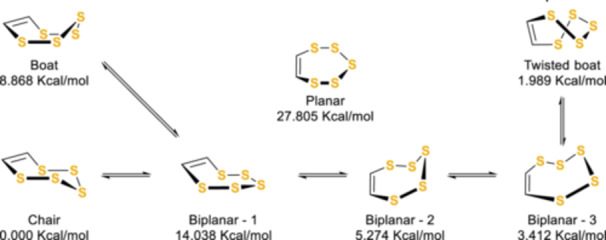
Conformational possibilities of the pentathiepin ring and Δ*E*, as studied by Buemi et al.^[^ ^79^ ^]^

There have been many crystallographic structures solved for pentathiepin derivatives, in part to investigate the exotic properties; interestingly, all of these structures have shown a chair‐like pentathiepin ring conformation.^[^ ^31^, ^72^, ^73^, ^75^, ^76^, ^77^, ^78^ ^]^ Indeed, the planar conformation of most polysulphide‐containing compounds is unfavorable, while the boat form and its derivatives are predicted to be ~8 Kcal/mol less stable than the chair. The calculations performed by Buemi et al. on the bare pentathiepin functionality demonstrated that the chair conformation appeared to be a semi‐rigid structure and that the boat‐chair interconversion seemed to be improbable and would have to cross through one of the high‐energy biplanar transition states (Scheme 17).^[^ ^79^ ^]^

**Scheme 18:**
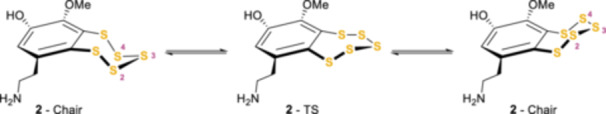
Inversion of the pentathiepin ring in compound **2**.

Subsequent to the discovery of varacin and related natural benzopentathiepins, there were extensive studies to rationalize the structures of these compounds. Two independent research groups in 1994 reported unexpectedly complex signals in the ^1^H‐NMR spectra of varacin and lissoclinotoxin A for the benzylic protons of their side chains.^[^ ^70^, ^71^ ^]^ Indeed, increasing the temperature from 25°C to 100°C led to the sharpening of these signals, with the observation of distinct diasterotopic signals. The sulfur atoms S2, S3, and S4 are either above the plane of the benzene ring or below, while the substituents on the benzene ring eliminate a potential plane perpendicular to that ring. The origin of this behavior has been explained by a slow inversion of the pentathiepin ring due to an exceptional energy high barrier (~29 Kcal/mol) to ring inversion, which ultimately led to asymmetry in the molecule and chirality (Scheme 18).^[^ ^76^ ^]^ In theory, this barrier should be sufficiently high to allow for the isolation of the pure enantiomers.

**Scheme 19:**
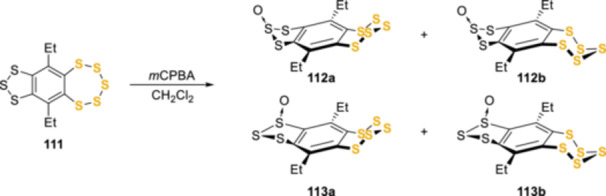
Structures of the four monoxides obtained by Kimura et al.

Kimura et al. published a series of work related to this pursuit, focused on the preparation, isolation, and characterization of enantiomerically pure 6,10‐diethyl[1,2,3]trithiolo[*h*]benzopentathiepins monoxides **112a**,**b** and **113a**,**b**, obtained by the oxidation of the trithiolobenzopentathiepin **111** with *m*CPBA (Scheme 19).^[^ ^72^, ^73^, ^78^ ^]^ Compounds **112a** and **112b** were conformational isomers with respect to the pentathiepin ring, whereas **113a** and **113b** were diastereomers with respect to the conformation of the pentathiepin ring and the configuration of the sulfinyl sulfur atom, respectively. Interestingly the corresponding pairs of oxides interconverted slowly at room temperature in chloroform, by inversion of the pentathiepin ring. The activation parameters of the isomerization were determined by means of ^1^H‐NMR spectroscopy and resulted in a barrier close to 24 Kcal/mol. Nonetheless, enantiomeric enrichment in natural pentathiepins has not been confirmed and optical stability has not been observed. The investigation of the chair–chair interconversion of natural pentathiepins is pivotal to understand both their biosynthesis and properties. The biosynthesis may favor one enantiomer over the other, but to date, this compound class has always been observed and isolated as racemates.^[^ ^80^ ^]^

**Scheme 20:**
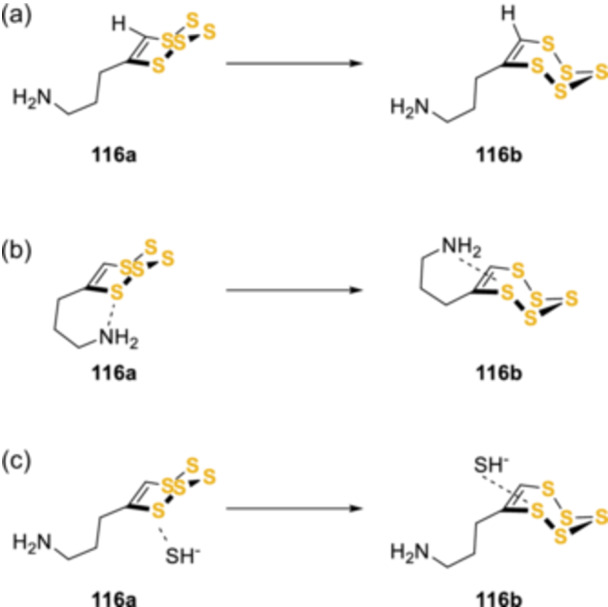
Racemization studies performed by Brzostowska et al.

Pentathiepin structures and reactions have been studied in detail using density functional theoretical (DFT) calculations by Greer et al.^[^ ^81^ ^]^ The 3‐[1,2,3,4,5]‐pentathiepin‐6‐yl‐propylamine (**116**, Scheme 20) was chosen as the model unsymmetrical pentathiepin. Consistent with the previously mentioned reports, there was a high energy barrier associated with the conversion of enantiomers **116a** to **116b** (Scheme 20a), this was confirmed at the B3LPY/6‐31 G(d) level. Similar results were obtained for the racemization induced by the intramolecular nucleophilic attack by the side‐chain amine to the pentathiepin ring (Scheme 20b). While a different low‐energy pathway for pentathiepin racemization was computationally predicted in the presence of HS^‐^ nucleophile, and chosen as a model for biological nucleophiles including glutathione (GSH) (Scheme 20c). A low‐energy path to thiolate ion‐assisted racemization suggested a potential easy loss of planar chirality, leading to difficulties in the study of enantiospecific behaviors such as the toxicity difference between the two conformations. Moreover, the ease of thiolate ion attack on the pentathiepin ring could also suggest that other nucleophiles may induce optical instability on the laboratory time scale. This possibility had been previously computationally and experimentally explored by the same team,^[^ ^68^, ^82^ ^]^ when they suggested that the decomposition of the pentathiepin ring could be triggered by a nucleophilic attack by either HS^‐^ or an intramolecular primary or secondary amine. Intriguingly, according to the computed reaction pathway, the energy barrier of a nucleophilic attack by a primary or secondary amine at S1 of the pentathiepin ring was 4.9 Kcal/mol lower than an attack at S2, leading to a more favorable extrusion of S_3_ rather than S_2_. This hypothesis was partially supported by experimental trapping of expelled sulfur species with norbornene and dimethylbutadiene. In particular, the loss of S_3_ could occur thermally and, to a small extent, with diethylamide ion in benzene/DMF mixture (10:3) or with free secondary amine in water/acetonitrile mixture (4:1).^[^ ^68^ ^]^

**Scheme 21:**
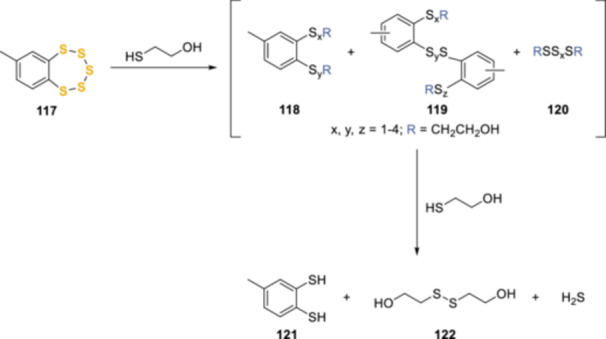
Investigation of the reaction between model benzopentathiepin and 2‐mercaptoethanol.

The reaction between thiols and pentathiepin rings has also been experimentally demonstrated by Chatterji et al., treating 7‐methylbenzopentathiepin **117** (Scheme 21) with moderate and high concentrations of 2‐mercaptoethanol. The products obtained with three equivalents of thiol were polysulfides (di‐, tri‐, tetra‐, penta‐, and hexasulfides) derived from the thiol itself and various aromatic polysulfide species derived from **117**. When a large excess of thiol was used, a less complex product mixture was obtained, mainly composed of **121**, **122**, and hydrogen sulfide as major products. Moreover, under physiologically relevant conditions, the half‐life of **117** in the presence of glutathione in buffered aqueous solution at 25°C was less than 1 min.^[^ ^83^ ^]^

## 5 BIOLOGICAL APPLICATION OF PENTATHIEPINS

**Figure 2:**
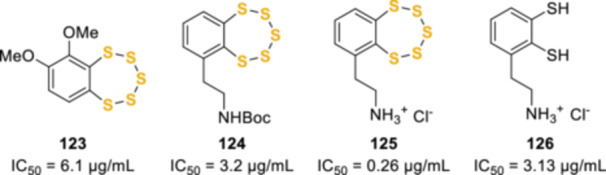
Benzopentathiepins endowed with cytotoxic activity against the HeLa53 cell line.

The investigations around the pentathiepin reactivity were principally aimed to investigate and explore the possibility to use pentathiepins in therapeutic applications. The methanolic extract of *Lissoclinum perforatum*, containing lissoclinotoxin A (**2**), isolissoclinotoxin A (**3**), and lissoclinotoxin B (**7**), demonstrated good potency against *Staphylococcus aureus* and *Escherichia coli*, with inhibition zones in a disk diffusion assay of 35 and 24 mm at 300 μM, respectively, and cytotoxicity against L1210 leukemia cell line (ID_50_ 3.5 μg/mL).^[^ ^5^ ^]^ A subsequent investigation at Smith Kline Beecham with extracts from *Lissoclinum japonicum*, collected off the coast of Palau, demonstrated that other metabolites of the 5‐(methylthio)varacin family, namely **5** and **6**, were PKC inhibitors with IC_50_ values of 3.0 and 0.3 μM/mL, respectively, with good selectivity over protein kinase A (PKA) (IC_50_ > 50 and 25 μM/mL, respectively). This study also found, in another extract from *Eudistroma* spp. off the coast of Pohnpei, the di‐phenolic compound 3,4‐desmethylvaracin (**4**), which also displayed potent inhibition of 0.5 μM/mL against PKC.^[^ ^6^ ^]^ Moreover, *N*,*N*‐dimethyl‐5‐(methylthio)varacin **6** and the synthetic benzopentathiepins **123**, **124**, and **125** (Figure 2) also demonstrated cytotoxicity toward HeLa53 cells with IC_50_ 6.1, 3.2, and 0.26 µg/mL, respectively. Interestingly, pentathiepin **125** is 10 times more cytotoxic than the corresponding *ortho*‐dithiol **126**.^[^ ^84^ ^]^

**Figure 3:**
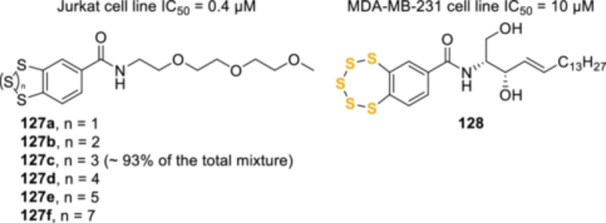
PEGylated and ceramide‐conjugated benzopentathiepins.

Varacin itself, first extracted from *Lissoclinum vareau*, exhibited potent antifungal activity against *Candida albicans* (14 mm zone of inhibition at 2 µg of varacin/disk) and cytotoxicity toward the human colon cancer HCT116 (IC_90_ = 0.05 µg/mL), 100 times the activity of 5‐fluorouracil (5‐FU) in the same assay.^[^ ^3^ ^]^ Varacin has also been shown to cleave single‐stranded DNA, *via* a redox pathway at pH 5.0 and 5.5. In the same study, the cytotoxic activity of **1** was evaluated on eight different human cancer cell lines, with IC_50_ values ranging from 0.60 to 48.40 nM.^[^ ^85^ ^]^ These data were consistent with the previously mentioned studies on the thiol‐dependent DNA‐cleaving ability of 7‐methylbenzopentathiepin (**117**). The authors also suggested that the presence of an aminoethyl substituent in all‐natural benzopentathiepins, which probably exists almost exclusively in the charged (‐NHR_2_^+^) form under physiological conditions, could increase the DNA affinity of these compounds.^[^ ^83^, ^86^ ^]^ A series of later studies demonstrated that the presence of the amino sidechain was not necessary for the antiproliferative activity. Mahendran et al. synthesized benzopolysulphanes with different sulfur‐ring sizes (**127a**–**f**, Figure 3) and a polyethylene glycole (PEG) group attached to the 4‐position of the aromatic ring, through an amide bond. The mixture of these polysulphanes, among which benzopentathiepin **126c** was the major component (93%), displayed increased antiproliferative activity compared with the unsubstituted benzopolysulphanes.^[^ ^87^ ^]^ Similar results were obtained several years later, by conjugating the benzopentathiepin core to a ceramide moiety (**128**).^[^ ^88^ ^]^

**Figure 4:**
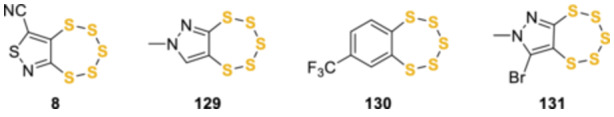
E. I. Du Pont de Nemours key anti‐fungal compounds.

Despite lacking a sidechain amino group, a series of very potent fungicidal pentathiepins (**8**, **129**–**131**, Figure 4) had been patented by E. I. Du Pont de Nemours in the late 1980s. The series was exemplified by **8** and included 7‐methyl‐7*H*‐[1,2,3,4,5]pentathiepino[6,7‐*c*]pyrazole **129**, which provided near total control of common fungi, including Apple Scab (100%), Tomato Late Blight (99%), Cucumber Powdery Mildew (99%), Wheat Rust (100%), Bean Rust (100%), and Beet Cercospora (85%), which was similar to other cysteine reactive heterocycles, including dithiazoles.^[^ ^89^ ^]^ Several enhancements were made in the mid‐1980s with a new series of compounds with the best being 7‐(trifluoromethyl)benzo[*f*][1,2,3,4,5]pentathiepin **130** and 8‐bromo‐7‐methyl‐7*H*‐[1,2,3,4,5]pentathiepino[6,7‐*c*]pyrazole **131**.^[^ ^7^, ^8^, ^9^, ^10^ ^]^

**Figure 5:**
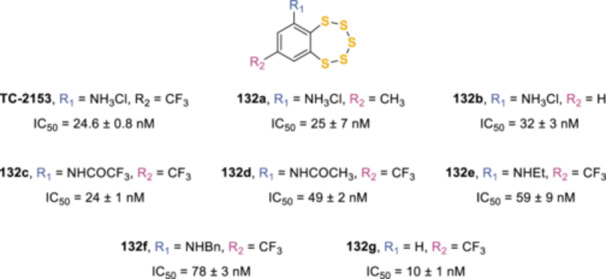
Structure–activity relationships (SAR) studies of TC‐2153 analogs as striatal‐enriched protein tyrosine phosphatase (STEP) inhibitors.

Then, in the late 2000s several new synthetic methods produced refreshed interest in the pentathiepin core. In particular, Khomenko et al.^[^ ^36^ ^]^ treated dithiolone **45** with sodium hydrosulphide hydrate in DMSO followed by hydrochloric acid, generating 8‐(trifluoromethyl)‐1,2,3,4,5‐benzopentathiepin‐6‐amine, the free‐base form of **9** (Figure 1). This compound was shown to completely block the development of pentylenetetrazole (PTZ)‐induced convulsions and to decrease mortality from nicotine‐induced convulsions when intragastrically administered at a dose of 10 mg/kg to outbred white mice. Moreover, it also displayed a marked anxiolytic effect, with a combined low level of acute toxicity with LD_50_ values of >1000 mg/kg. Another study^[^ ^90^ ^]^ correlated this anxiolytic and anticonvulsant activity with the expression of some serotonin‐related genes in the mouse brain. It was shown that chronic administration of the hydrochloride form **9**, namely TC‐2153, downregulated the expression of the serotonin 1 A receptor (5‐HT_1A_) and the major serotonin‐degrading enzyme, monoamine oxidase A (MAO‐A). Other studies also reported antidepressant activity for TC−2153 on AKR and D13 mice in the forced swim test, with no negative side effects on locomotor activity, anxiety, exploration, coordination and balance, and obsessive–compulsive‐like behavior. Similar promising results were obtained from another animal model in the “novel tank” test for psychotropic activity on *Danio rerio*.^[^ ^91^, ^92^, ^93^ ^]^ In 2012, it was demonstrated that TC−2153 possessed a broad spectrum of antinociceptive effects, expressing high analgesic activity both in the visceral pain test and thermal pain test. Treatment of animals with naloxone 10 min before testing completely blocked the analgesic activity of TC‐2153, thus suggesting that its effect was mediated by the opioidergic system.^[^ ^94^ ^]^ In 2014, elemental sulfur was discovered in a high throughput screen against STEP, a brain‐specific protein tyrosine phosphatase (PTP), implicated in many neurodegenerative diseases, including Alzheimer's disease.^[^ ^95^, ^96^ ^]^ The authors then sought to identify more conventional inhibitor structures that would improve solubility and enable further refinement. The benzopentathiepin core was chosen and TC‐2153 was selected and identified as sulfur surrogate, due to its reasonable water solubility, low toxicity, and ability to cross the blood–brain barrier. TC‐2153 inhibited STEP with IC_50_ of 24.6 nM and increased the tyrosine phosphorylation of three STEP substrates in intact neurons in culture and in vivo in the cortex of wild‐type (WT) mice while possessing good in vitro and in vivo selectivity over other PTPs. In the same work, the authors also assessed the mechanism of action of TC‐2153, suggesting an irreversible inhibition of the enzyme by covalent interaction with the catalytic cysteine‐472.^[^ ^11^ ^]^ Alzheimer's disease mouse models were employed by several research groups to demonstrate the efficacy of TC‐2153 in reversing cognitive deficits and altered nociception.^[^ ^11^, ^97^, ^98^ ^]^ Recent work by Rudnitskaya et al.^[^ ^99^ ^]^ showed some ambiguity on the behavior of the animals following chronic administration of TC‐2153, which was shown to reduce the already low locomotor activity and to enhance anxiety‐related behavior in OXYS rats, while improving their long‐term memory in the Morris water maze. More recently, it was also suggested that an abnormal STEP signaling pathway was involved in sepsis‐associated encephalopathy (SAE), a potentially irreversible acute cognitive dysfunction invoked by the immune response to endotoxic bacterial cell wall components. Notably, TC‐2153 treatment alleviated sepsis‐induced memory impairment by increasing phosphorylation of GluN2B and ERK1/2, CREB/BDNF, and PSD95.^[^ ^100^ ^]^ The pentathiepin core offered a totally new class of compounds against this target phosphatase super family.^[^ ^101^ ^]^ Indeed, a series of TC‐2153 analogs (**132a**–**g**, Figure 5) were prepared to investigate the structure–activity relationships (SARs) of this class of compounds, specifically regarding the trifluoromethyl and amino substitutions. This study confirmed the activity of TC‐2153 (IC_50_ = 24.6 ± 0.8 nM) and showed the possibility to either modify, as in **132a**, or remove, as in **132b**, the trifluoromethyl group in 8‐position of the benzopentathiepin ring (IC_50_ = 25 ± 7 nM and 32 ± 3 nM, respectively). Modifications of the aniline moieties highlighted the importance of an electron‐withdrawing substituent to maintain the activity, as shown by the decreasing potencies of the trifluoroacetamide **132c** (IC_50_ = 24 ± 1 nM), acetamide **132d** (IC_50_ = 49 ± 2 nM), and secondary amines **132e**,**f** (IC_50_ = 59 ± 9 nM). Interestingly, the most potent compound of the series was the simple 7‐(trifluoromethyl)‐benzopentathiepin **132 g** (IC_50_ = 10 ± 1 nM), but the absence of the amine substituent hampered its solubility and removed a potentially useful site for introducing reporter groups or functionalities for pull down and proteomic applications.^[^ ^102^ ^]^

Further studies expanded TC‐2153 applications to the attenuation of fear‐induced aggression and aggressive reactions in rats.^[^ ^103^, ^104^ ^]^ Chatterjee et al. also showed a therapeutic benefit in a fragile X syndrome mouse model, by reversing audiogenic seizure incidences, reducing hyperactivity and anxiety states, and increasing sociability.^[^ ^105^ ^]^

**Figure 6:**
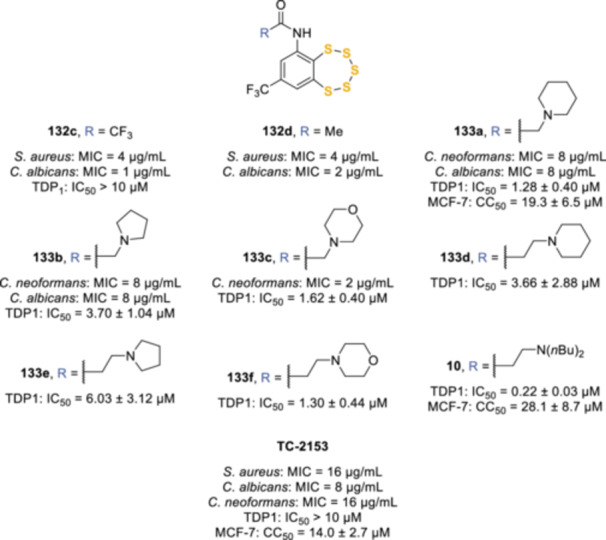
TC‐2153 derivatives endowed with antimicrobial and tyrosyl‐DNA phosphodiesterase 1 (TDP1) inhibitory activity.

More recently, TC‐2153 was investigated for its antimicrobial activity, together with a series of known and novel amide derivatives (**132c**,**d**, **133a**–**f**, Figure 6). TC‐2153 showed moderate antibacterial activity against *Staphylococcus aureus*, with a MIC of 16 μg/mL, while the trifluoroacetamide **132c** and acetamide **132d** resulted in a four‐fold increase in the activity, with the MIC reaching 4 μg/mL, and potent inhibition of *Candida albicans* (MIC of 1 and 2 μg/mL, respectively). Other derivatives bearing a cyclic tertiary amine (**133a**–**f**) were less potent antimicrobial agents on *S. aureus* and *C. albicans* (MIC = 8 μg/mL), with **133c** displaying moderate activity against *Cryptococcus neoformans* (MIC = 2 μg/mL).^[^ ^106^ ^]^ All compounds containing the pentathiepin ring had no activity against Gram‐negative bacteria (MIC > 32 μg/mL). TC‐2153, **132d**, and **133a**–**c**, together with other amide analogs (**133d**–**f**, **10**), have also been investigated by the same research group as inhibitors of tyrosyl‐DNA phosphodiesterase 1 (TDP1).^[^ ^12^ ^]^ This DNA repair enzyme is implicated in the removal of stalled topoisomerase 1 DNA covalent complexes (Top1‐DNAcc) that can be generated by some anticancer Top1 inhibitors, including topotecan and irinotecan, thus being a promising target for enhancing the therapeutic effect of anticancer Top1 inhibitors.^[^ ^107^, ^108^ ^]^ In this case, TC‐2153 and the trifluoroacetamide **132c** (IC_50_ > 10 μM for both compounds) were weaker inhibitors of TDP1 compared with the cyclic tertiary amine‐bearing analogs **133a**–**133f** (IC_50_ between 1.28 and 6.03 µM). Interestingly, the most potent compound of the series was **10**, which possessed a more flexible acyclic tertiary amine chain and displayed a sub‐micromolar inhibitory activity (IC_50_ = 0.22 µM). Compounds **10** and **133a** also exerted moderate apoptotic cytotoxic activity on the MCF‐7 cancer cell line (CC_50_ values of 28.1 and 19.3 µM, respectively), albeit less potent than TC‐2153 (CC_50_ = 14.0 µM).

**Figure 7:**
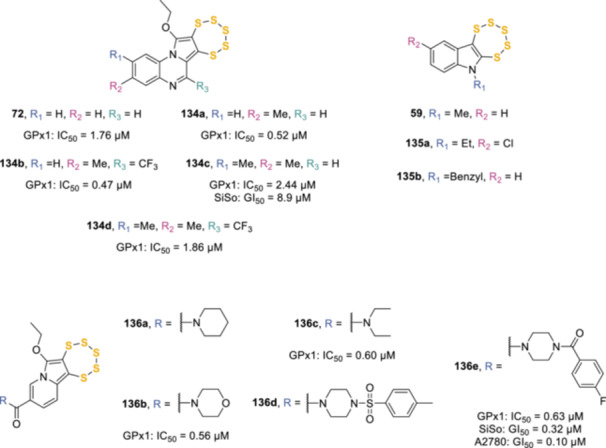
Pentathiepin‐based GPx1 inhibitors.

Behnisch‐Cornwell et al. synthesized a series of pentathiepins with various heterocyclic rings (**72**, **134a**–**d**, **59**, **135a**,**b**, Figure 7) endowed with submicromolar inhibitory activity against bovine erythrocyte glutathione peroxidases (GPx), a family of key antioxidative enzymes responsible for the intracellular destruction of H_2_O_2_ and organic peroxides. These compounds caused oxidative stress in SiSo human cervix cancer cells, resulting in DNA strand breaks, induction of apoptosis, and cell death.^[^ ^109^ ^]^ On the basis of its more distinct selectivity with regard to GPx1 inhibition and its slightly higher aqueous solubility, pyrrolo[1,2‐*a*]quinoxaline **134c**, was selected for further studies, which led to the preparation of five new indolizine‐based pentathiepins (**136a**–**e**, Figure 7) characterized by a nicotinamide backbone to potentially increase water solubility. The authors assessed the potential of the compounds to inhibit the isolated GPx1 and then screened for cytotoxicity across a panel of 14 human cancer cell lines under normoxic and hypoxic conditions. In selected cell lines, the induction of oxidative stress was analyzed, and subsequently, the DNA damaging potential was assessed. Particular emphasis was directed toward the cell death mechanism, with experiments covering the induction of apoptosis or ferroptosis. Three out of five pentathiepins (**136b**,**c**,**e**) had a strong inhibitory effect on the bovine erythrocyte GPx1, with IC_50_ values in the submicromolar range, and they also exhibited low or submicromolar IC_50_ values on several cancer cell lines.^[^ ^110^ ^]^ The best‐performing compound of the series (**136e**) suffered from poor water solubility and limited chemical stability in biological media, therefore it was formulated into 1,2‐dioleoyl‐*sn*‐glycero‐3‐phosphocholine liposomes and assessed for its chemical stability in the presence of GSH, water solubility, and antiproliferative activity on two human cancer cell lines. Gratifyingly, the solubility of the pentathiepin in aqueous media at a pH of 7.4 increased from practically insoluble to as high as 400 µM, while it showed a 4‐fold decrease in the initial degradation rate and time to complete degradation in the presence of 10 mM GSH. Liposomal **136e** also retained the antiproliferative activity against A2780 and SiSo cancer cell lines, with GI_50_ values of 0.09 and 0.29 µM, respectively, while the free pentathiepin showed GI_50_ values of 0.10 and 0.32 µM, respectively (as a comparison, cisplatin inhibited cancer cells growth with GI_50_ = 0.41 and 0.24, respectively). These features made liposomes promising vehicles for stabilizing pentathiepins in biological medium.^[^ ^111^ ^]^

**Figure 8:**
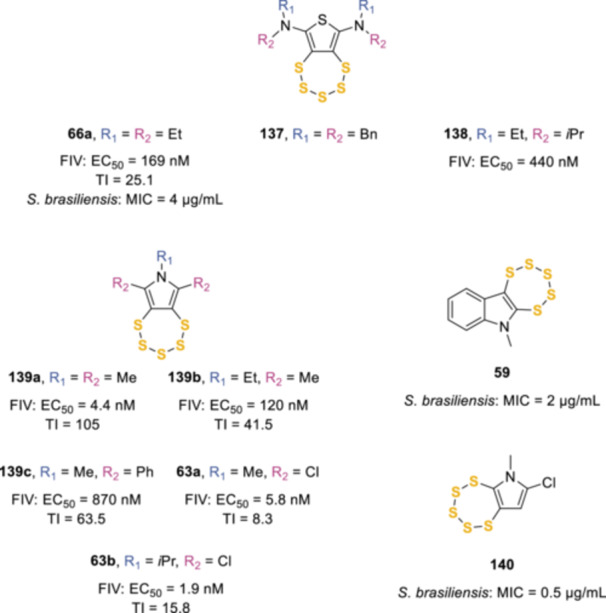
Antiviral and antifungal pentathiepin‐based agents.

As part of a wider medicinal chemistry program to find novel chemotypes for nucleocapsid protein (NCp) inhibitors, our research group investigated a set of dual‐functionality disulfide tetrathiocine derivatives as NCp inhibitors of the feline immunodeficiency virus (FIV), used as a model of human immunodeficiency virus (HIV) infection.^[^ ^67^ ^]^ The NCp contains the conserved double zinc finger peptide unit C–X_2_–C–X_4_–H–X_4_–C (CCHC) that is found in nearly all retroviruses, including HIV.^[^ ^112^ ^]^ Effective targeting of these zinc fingers would render the virus inert as deletion or modification of either zinc finger leads to virus inactivation. While there is also the possibility to directly compete with the nucleic acid binding site, the authors decided to interfere directly with the zinc finger structure *via* zinc ejection. In addition, one of the pentathiepin starting materials (**66a**, Scheme 8, Figure 8) was also tested, and it showed good activity (EC_50_ = 169 nM) against FIV viral loading, thus justifying further studies on this core.^[^ ^67^ ^]^ Indeed, in 2019 a deeper investigation of the pentathiepin functionality as a potential FIV inhibitor was published.^[^ ^113^ ^]^ Compounds **59**, **63a**,**b**, **66a**, **137**, **138**, **139a**–**c**, and **140** were initially tested at three high concentrations (1–100 µM) in a short MTT cell viability assay using CRFK cells to assess nonspecific toxicity, then they were tested against FIV using an interleukin‐2 (IL‐2‐) independent feline lymphoblastoid (FL‐4) cell line. Except for **137** (EC_50_ = 75 µM), the submicromolar potencies of these compounds, with EC_50_ values ranging from 4.4 nM for **139a** to 870 nM for **139c**, coupled with low to moderate toxicities, showed an interesting avenue of investigation toward the development of a candidate compound for targeting the FIV/HIV NCp. Compound **139c** was the most interesting result with slightly lower potency (EC_50_ = 870 nM) but significantly decreased toxicity (CC_50_ = 55 µM, TI = 63.5), suggesting that there is potential to reduce any nonspecific toxicity associated with the pentathiepin scaffold. A mechanism of action for these compounds was also proposed, which involved a zinc‐coordinating cysteine thiol(ate) reacting with the pentathiepin ring system at the S2–S3 bond to generate a transient protein–pentathiepin inserted bound unit. This then underwent a rearrangement to form an intramolecular protein disulfide with a consequent decrease in zinc ion affinity.^[^ ^113^ ^]^ These analogs were also screened against eight skin lesion isolates of *Sporothrix brasiliensis*, the causative agent of zoonotic sporotrichosis in Brazil.^[^ ^114^ ^]^ It was found that increasing the size of the amine substituent of the thiophene, from ethyl (**66a**) to benzyl (**137**), reduced the activity (MIC = 4 and > 8 µg/mL, respectively). Pyrroles **139a**–**c** were also inactive on all eight skin isolates, while compounds **63a**,**b** displayed some activity on two and three isolates respectively (MIC = 8 µg/mL). Interestingly, the unsymmetrical pentathiepins **59** and **140** showed promising antifungal activity across all isolates, with **140** presenting a lower MIC than itraconazole, which is the current standard of care for sporotrichosis, in four out of eight clinical isolates, and **59** demonstrating a dose‐dependent inhibition of *S. brasiliensis* growth, albeit being slightly less potent.^[^ ^115^ ^]^

**Figure 9:**
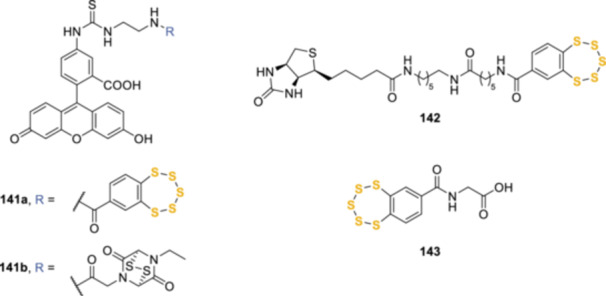
Benzopentathiepins applied to thiol‐mediated uptake studies.

Another interesting application of pentathiepins reactivity has been conducted by Matile et al., the authors reported pentathiepins have been able to efficiently mediate cell uptake through dynamic covalent dichalcogenide exchange. This mechanism, often referred to as thiol‐mediated uptake, takes advantage of exofacial thiol groups present on the cell surface to enhance cellular association and internalization of various molecules, such as peptides, oligonucleotides, nanoparticles, probes, fluorescent dyes, and so on, bearing thiol‐reactive moieties in their structures, such as cyclic disulfides and diselenides.^[^ ^116^, ^117^ ^]^ Pentathiepin **141a** (Figure 9) outperformed all the other known cyclic oligochalcogenides, being 10 and 140 times more active than the well‐studied epidithiodiketopiperazines and asparagusic acid, respectively. Most importantly, according to HPLC analysis combined with mass spectrometry, benzopentathiepin **141a** penetrated the cells in novel ways originated by their high reactivity and specificity toward exofacial thiols that led to the formation of adaptive dynamic‐covalent networks of extreme sulfur species, including cyclic oligomers with up to 19 sulfur atoms.

The potential applicability of this concept was proved by means of two different methods. In the first case, two biotin binding sites of the streptavidin tetramer were conjugated with biotinylated pentathiepin **142**, while the other sites were loaded with an artificial ruthenium‐based metalloenzyme able to cleave the allyloxycarbonyl protecting group on a rhodamine‐based fluorescent probe. Incubation of HeLa Kyoto cells first with the loaded streptavidin tetramer, then with the freely diffusing protected rhodamine, resulted in the intracellular emission from the deprotected fluorophore probe. This indicated that the benzopentathiepin‐bearing streptavidin had been efficiently internalized by the cells, while the control experiments using pentathiepin‐free streptavidin did not register any fluorescence. In the second scenario, the authors designed a cell‐penetrating streptavidin (CPS) by covalently linking a benzopentathiepin to streptavidin *via* copper‐catalyzed azide‐alkyne cycloaddition reaction. In this way, all four biotin‐binding sites of the CPS were available to load biotinylated fluorescein as a model substrate. Confocal laser scanning microscopy images of HeLa Kyoto cells incubated with fluorescein‐loaded CPS revealed intense diffuse fluorescence in most parts of the cells.^[^ ^118^ ^]^ In a follow‐up study, the authors demonstrated that CPS bearing four benzopentathiepin moieties were able to efficiently introduce within the cells a variety of different molecules, thus demonstrating the potential compatibility of this pentathiepin‐based tool with targeted delivery, interfacing of nanobodies, peptide nucleic acids, and mechanosensitive fluorescent flipper probes with HaloTag‐GFP (green fusion protein) fusion proteins, and their controlled release through intracellular desthiobiotin−biotin exchange.^[^ ^119^ ^]^

The same team explored the possibility to take advantage of benzopentathiepins high activities toward exofacial thiols to inhibit thiol‐mediated uptake, which has been confirmed to play an essential role in the cellular entry of viruses, such as HIV,^[^ ^120^ ^]^ and toxins, including diphtheria toxin.^[^ ^121^ ^]^ The authors used benzopentathiepin **141a** and epidithiodiketopiperazine **141b** as fluorescent cell‐penetrating reporters for the screening of thiol‐mediated uptake inhibitors of various natures. Among the chosen thiol‐reactive probes, pentathiepin **143** showed an outstanding inhibition against reporter **141b** (MIC = 0.3 µM), but also a moderate activity against the pentathiepin‐based reporter **141a** (MIC = 4 µM).^[^ ^122^ ^]^ The reactivity of benzopentathiepin **143** toward extracellular thiols was also explored as a tool to investigate the modulation of thiol‐mediated cell uptake of oligonucleotide phosphorothioates (OPS), that is, DNA or RNA mimics where one oxygen atom of the phosphate group has been replaced by a sulfur atom, increasing not only the oligonucleotides stability toward nucleases but also their hydrophobicity and cell penetration.^[^ ^123^ ^]^ The use of benzopentathiepin **143** as an inhibitor of thiol‐mediated uptake interfered with the cell penetration of OPS, thus hinting toward this mechanism as a way for OPS internalization. Interestingly, the complementary incubation of **143** with OPS was shown to trigger the emergence of an adaptive dynamic polysulfide network that facilitated the exchange with cell surface thiols and thus activated OPS thiol‐mediated uptake.^[^ ^124^ ^]^ Very recently, a first attempt of mechanistic investigation has been performed by the same team that evaluated the uptake of fluorescent probes into HeLa Kyoto cells by means of automated high‐content high‐throughput (AHCHT) microscopy. In this work, transporter **141a** and inhibitor **143** were used to study thiol‐mediated uptake and the possible proteins involved in the interaction with these cell‐penetrating sulfides. The fluorescence intensities were measured in three different situations, namely, (i) normal condition, where no modification to the system was applied, (ii) knockdown of the putative exchange partner proteins, and (iii) presence of alternative inhibitors of these proteins. The results suggested the engagement of protein disulfide‐isomerase A3 (PDIA3), a multifunctional protein observed at the cell surface and involved in many processes, such as signal transduction, translocation, viral entry, and redox homeostasis,^[^ ^125^, ^126^ ^]^ in the thiol‐mediated uptake of benzopentathiepins.^[^ ^127^ ^]^

## 6 CONCLUSIONS

Since their first synthesis in the laboratory in the 1960s and 1970s, pentathiepins have attracted chemists’ interest for their peculiar chemical properties, uncommon stability, chiral behavior, and useful reactivity. However, they rarely drew the attention of medicinal chemists and biologists, with the notable exception of some potent antifungal pentathiepins patented by E. I. Du Pont de Nemours in the 1980s. This only partially changed in 1991 when the first natural products bearing a pentathiepin ring were discovered. Varacin and its related metabolites displayed potent biological activities, ranging from cytotoxicity against cancer cell lines to PKC inhibition to anti‐bactericidal properties. The next decade was fully devoted to the development of novel and robust approaches to the preparation of both natural and synthetic analogs, together with in‐depth investigations, both theoretical and experimental, aimed at unveiling their structures and conformations. The interest of the scientific community waned, but novel synthetic approaches continued to be developed, and further biological activity discoveries were made. The key discovery of TC‐2153 as a sulfur isosteric replacement breathed new life into the field, proving to be a surprisingly simple compound with an exceptionally wide range of biological activities both in vitro and in vivo. The unique reactivity of the pentathiepin ring, coupled with the interesting pharmacokinetic properties of the 6‐amine and 8‐trifluoromethyl substituents, allowed TC‐2153 to exert potent anticonvulsant, anxiolytic, antidepressant, and antinociceptive activities with reduced side effects on the locomotor system and low toxicity. These promising properties led Xu et al. to choose pentathiepins, particularly TC‐2153, as STEP inhibitors in place of the serendipitously discovered elemental sulfur, thus proving the versatility of this class of heterocycles and expanding the range of their biological activities to neuroprotection. TC‐2153 and its analogs, together with different classes of pentathiepin‐containing compounds have proven to be potent anticancer, antibacterial, antifungal, and antiviral agents. The instability of the pentathiepin moiety toward biologically relevant reactive species, such as glutathione, has been highlighted, but the possibility to modulate its reactivity by different substituents has allowed the development of potently active compounds in in vivo models. The pentathiepin ring systems reactivity has also been exploited as a transporter utilizing exofacial thiols to enhance the internalization of a wide variety of peptides, oligonucleotides, nanobodies, probes, and so on through thiol‐mediated uptake. Pentathiepins have been widely investigated for their reactivity and unique structural properties, and the reported examples clearly highlight the potential of pentathiepin medicinal chemistry. The intermittent developments in the field, have left substantial room to develop and increase the knowledge and the biological applications of this promising but still understudied moiety. These representative examples prove that despite its unusual structure the pentathiepin has a wide tuneable repertoire to offer the medicinal chemistry community and to the medicinal chemist's toolbox.

## CONFLICTS OF INTEREST STATEMENT

The authors declare no conflicts of interest.

## Data Availability

Data sharing is not applicable to this article as no new data were created or analyzed in this study.
